# Conformational Changes in a Macrolide Antibiotic Binding Protein From *Mycobacterium smegmatis* Upon ADP Binding

**DOI:** 10.3389/fmicb.2021.780954

**Published:** 2021-12-09

**Authors:** Qingqing Zhang, Xiang Liu, Huijuan Liu, Bingjie Zhang, Haitao Yang, Kaixia Mi, Luke W. Guddat, Zihe Rao

**Affiliations:** ^1^State Key Laboratory of Medicinal Chemical Biology, Frontiers Science Center for Cell Responses, College of Life Sciences, Nankai University, Tianjin, China; ^2^Innovative Center for Pathogen Research, Guangzhou Laboratory, Guangzhou, China; ^3^CAS Key Laboratory of Pathogenic Microbiology and Immunology, Institute of Microbiology, Chinese Academy of Sciences, Beijing, China; ^4^Shanghai Institute for Advanced Immunochemical Studies and School of Life Sciences and Technology, ShanghaiTech University, Shanghai, China; ^5^School of Chemistry and Molecular Biosciences, The University of Queensland, Brisbane, QLD, Australia; ^6^Laboratory of Structural Biology, School of Life Sciences and School of Medicine, Tsinghua University, Beijing, China

**Keywords:** crystal structure, conformational change, macrolide antibiotic binding protein (MABP), non-canonical ABC transporter, macrolide antibiotic, erythromycin

## Abstract

Rv3197 (MABP-1), a non-canonical ABC protein in *Mycobacterium tuberculosis*, has ATPase activity and confers inducible resistance to the macrolide family of antibiotics. Here we have shown that MSMEG_1954, the homolog of Rv3197 in *M. smegmatis*, has a similar function of conferring macrolide resistance. Crystal structures of apo-MSMEG_1954 (form1 and form 2) and MSMEG_1954 in complex with ADP have been determined. These three structures show that MSMEG_1954 has at least two different conformations we identify as closed state (MSMEG_1954-form 1) and open state (MSMEG_1954-form 2 and MSMEG_1954-ADP). Structural superimposition shows that the MSMEG_1954-form 2 and MSMEG_1954-ADP complex have similar conformation to that observed for MABP-1 and MABP-1-erythromicin complex structure. However, the antibiotic binding pocket in MSMEG_1954-form 1 is completely blocked by the N-terminal accessory domain. When bound by ADP, the N-terminal accessory domain undergoes conformational change, which results in the open of the antibiotic binding pocket. Because of the degradation of N terminal accessory domain in MSMSG_1954-form 2, it is likely to represent a transitional state between MSMEG_1954-form 1 and MSMEG_1954-ADP complex structure.

## Introduction

Tuberculosis (TB) is an infectious disease that is one of the top 10 causes of death worldwide (World Health Organization (WHO), 2018). Globally, an estimated 1.7 billion people are thought to be infected with *Mycobacterium tuberculosis* (MTB) (World Health Organization (WHO), 2019). Of great concern is the increasing prevalence of multidrug-resistant tuberculosis (MDR-TB) and extensively drug-resistant tuberculosis (XDR-TB), rendering many of the current treatments ineffective (World Health Organization (WHO), 2014, 2016). To begin to overcome this problem it is essential to have a comprehensive understanding as to how drug resistance has been developed. Genetic resistance to an anti-tuberculosis drug arises from spontaneous chromosomal mutations at low frequency (Zhang and Yew, 2009, 2015). Clinical drug-resistant TB largely occurs as a result of man-made selection during the treatment of these genetic alterations. *Mycobacteria* has developed diverse strategies to acquire resistance to the most widely used antibiotics (Alekshun and Levy, 2007; Goldberg et al., 2012; Blair et al., 2015), including antibiotic target protection by site-of-action mutations (Vilcheze and Jacobs, 2014) or by chemical modification of the target; the direct modification or inactivation of antibiotics by specific enzymes (e.g., hydrolases, phosphotransferases); the reduced cell-wall permeability (Goude and Parish, 2008) or increased efflux of the drugs (Braibant et al., 2000; De Rossi et al., 2006; da Silva et al., 2011; Black et al., 2014), which prevents the drug from gaining access to the target. ATP-binding cassette (ABC) families are important mediators of drug resistance in micro-organisms. Their function is to pump drugs out of the cell, thereby rendering them ineffective.

In our previous study, we have shown MTB has evolved a non-canonical ABC protein (type-II ABC proteins), Rv3197, identified as macrolide antibiotic binding protein-1 (MABP-1), which confers inducible resistance to the macrolide family of antibiotics (Zhang et al., 2018). This protein family is widely prevalent in other Mycobacteria (e.g., *Mycobacterium bovis* and *Mycobacterium smegmatis*, *Mycobacterium kansasii, and Mycobacterium avium*). Here, we show that MSMEG_1954, which has 79% sequence identity with MABP-1 (Supplementary Figure 1), is responsible for macrolide antibiotic resistance in *M. smegmatis*. Structures reported here, reveal substrate bound and apo-states of the protein, possibly associated that conformational changes upon substrate binding.

## Materials and Methods

### Bacterial Strains and Culture Conditions

As previously described (Zhang et al., 2018), cultures of *M. smegmatis* strains were grown in Middlebrook 7H9 medium (Becton Dickinson) supplemented with 0.5% (v/v) glycerol, 0.05% (v/v) Tween 80 (Sigma) and 10% ADS enrichment (5% (w/v) bovine serum albumin fraction V, 2% (w/v) dextrose and 8.1% (w/v) NaCl). Single bacterial colonies of *M. smegmatis mc^2^155* strain were cultured on Middlebrook 7H10 medium (Becton Dickinson) containing 10% ADS, and 0.5% (v/v) glycerol. Kanamycin, erythromycin and clarithromycin were purchased from Sigma.

### Generation of MSMEG_1954 Overexpressing Strain in *M. smegmatis*

To overexpress MSMEG_1954 in mycobacteria, the full-length coding gene of *msmeg_1954* was amplified from *M. smegmatis* genomic DNA and cloned into the mycobacteria overexpression vector pMV261 (Stover et al., 1991) to yield pMV261-*1954* for transformation into *M. smegmatis* (identified as pMV261-*1954*-mc^2^155). For a control, the empty pMV261 vector was also transformed into *M. smegmatis* and referred to as pMV261-mc^2^155.

### Antibiotic Resistance Testing

The inhibition curves under erythromycin treatment were determined in 50 ml 7H9 medium culture of recombinant *M. smegmatis* strains in 100 ml flasks, by monitoring OD_600_, as described previously (Li et al., 2015). Briefly, logarithmic phase *M. smegmatis* cultures (OD_600_ of 0.5) of tested strains were diluted to an OD_600_ of 0.1 and then allowed to grow in the presence or absence of 3 mg/L erythromycin at 37°C with shaking at 200 rpm. Cultures were monitored by measurement of OD_600_. The growth of recombinant *M. smegmatis* in the presence of 0.75 mg/L clarithromycin was measured after 24 h incubation. All antibiotic resistant tests were repeated at least three times. Statistical analyses were performed using *t*-tests. *** *p* < 0.001, ** *p* < 0.01, and *p < 0.05.

Survival under erythromycin treatment was determined as indicated. Logarithmic phase cultures (OD600 ∼ 0.3) were treated with 25 mg/L erythromycin for 3 h, serially diluted (1:10) and spotted (3 μL) onto 7H10 medium. Photographs were taken after 3 days of incubation at 37°C. Experiments were repeated at least three times.

### Quantitative Real-Time Polymerase Chain Reaction Analysis

Logarithmic phase cultures (OD_600_ of 0.8) of *M. smegmatis* strains were treated with different concentrations of erythromycin for 30 min and collected for RNA isolation, while untreated stains were set up as control groups. The corresponding cell pellets were resuspended into TRIzol (Invitrogen, United States) and RNA was purified according to the manufacturer’s instructions. SuperScript^TM^ III First-Strand Synthesis System (Invitrogen, United States) was used for cDNA synthesized. Quantitative real-time PCR (qRT-PCR) was performed using 2 × SYBR real-time PCR pre-mix (Takara Biotechnology Inc., Japan) on a Bio-Rad iCycler. *M. smegmatis rpoD* (the coding sequencing of RNA polymerase sigma factor SigA) was used to normalize the gene expression of *msmeg_1954*. The relative gene expression was calculated using the 2^–ΔΔCT^ method (Livak and Schmittgen, 2001). Experiments were repeated at least three times. Levels of mRNA expression of *msmeg_1954* under clarithromycin treatment was carried out in the logarithmic phase cultures (OD_600_ of 1.8) of *M. smegmatis* as described above.

### Cloning, Expression and Purification of MSMEG_1954 in *E. coli*

The gene encoding the full length *msmeg_1954* was amplified by polymerase chain reaction (PCR) from *M. smegmatis* genomic DNA and sub-cloned into prokaryotic expression vector pGEX-6p-1 with an N-terminal GST tag. Recombinant plasmid was transformed into *E. coli* BL21-Codon Plus (DE3) RIL for protein expression.

Cells were grown in Luria Bertani medium supplemented with 100 mg/L ampicillin at 37°C until OD_600_ reached 0.8 and then induced with 0.5 mM isopropyl-β-d-thiogalactopyranoside (IPTG) for 18 h at 16°C. Cells were harvested by centrifugation and lysed in 1 x PBS, pH 7.4 by ultrasonication. After centrifugation of cell lysates, supernatants containing soluble recombinant protein were loaded onto a GST-affinity chromatography (GE Healthcare) and then washed with 1 × PBS to remove non-specifically bound proteins. The proteolytic cleavage of the GST-tag was performed with PreScission Protease overnight in cold room, and the tagless MSMEG_1954 was further purified with a Superdex 200 (10/300) gel filtration column (GE Healthcare) pre-equilibrated with a buffer containing 25 mM Tris-HCl, pH 8.0, and 150 mM NaCl. Fractions containing target protein were then pooled and concentrated for crystallization. The expression and purification of MSMEG_1954 mutants used in the ATPase assay were carried out similarly as described above.

### Crystallization and Structure Determination

For crystallization, the purified MSMEG_1954 was concentrated to 20 mg/mL in 25 mM Tris-HCl (pH 8.0), 150 mM NaCl. The protein solution for Apo structure contained 20 mg/ml MSMEG_1954. To prepare the protein-ligand complex, MSMEG_1954 (20 mg/ml) and each ligand (protein: ligand molar ratio of 1: 5) were incubated overnight at 4°C before co-crystallization.

Initial crystallization screening was performed using sitting-drop vapor-diffusion at 293K and crystal screening kits from Hampton Research and Wizard from Rigaku. After about 1 month, crystals of apo-MSMEG_1954 (form 1) were obtained with reservoir solutions containing 0.2 M Ammonium acetate, 0.1 M Hepes (pH7.5), 25% w/v PEG3350. Crystals of apo-MSMEG_1954 (form 2) were obtained with a reservoir solution containing 0.2 M ammonium sulfate, 0.1 M Tris (pH8.5), 25% w/v PEG3350 after about 2 weeks. Crystals in complex with ADP grew in a solution containing 0.2 M calcium acetate hydrate, 0.1 M sodium cacodylate trihydrate (pH6.5), 18% PEG 8000. All crystals were soaked in the reservoir solution supplemented with 25% (v/v) glycerol before being flash-cooled in liquid nitrogen.

Diffraction data for form 1 crystals were collected on Beamline 17U of Shanghai Synchrotron Radiation Facility (SSRF) at 100K. Diffraction data for MSMEG_1954-ADP complex were collected on Beamline 18U of SSRF. Data were processed with the HKL2000 suite of programs (Otwinowski and Minor, 1997). Diffraction data for form 2 crystals were collected on beamline 19U of SSRF at 100 K and were integrated, scaled and merged using the HKL3000 (Minor et al., 2006).

The structure of form 1 and form 2 crystals was solved by molecular replacement (MR) with Phaser (McCoy et al., 2007) in CCP4 (Potterton et al., 2003) using the kinase domain of MABP-1 as a search template. The initial model was rebuilt by Phenix.AutoBuild (Adams et al., 2002). Manual adjustment of the model with Coot (Emsley et al., 2010) and refinement with Phenix located 395 residues and 293 waters in the model of form 1. In form 2, the electron density map of residues 1–97; 182–184, and 376–392 were not visible, and were therefore not included in the final structure. The MSMEG_1954-ADP complex structure was solved by molecular replacement (MR) using the form 2 crystal structure as a search template.

All structures were validated by MolProbity (Chen et al., 2010). The statistics of the phasing and refinement are summarized in Supplementary Table 1. The figures were prepared using PyMOL (DeLano Scientific) unless otherwise stated.

### Construction of MSMEG_1954 Point Mutants and an N-Terminal Truncation

Site-directed mutagenesis was performed using Fast Mutagenesis System (Trans Gen Biotech) following the manufacturer’s instructions. The plasmids pMV261- *msmeg_1954* and pGEX-6p-1-*msmeg_1954* were used as the corresponding template for constructing a series of mutants for overexpression in *M. smegmatis* and for protein purification in *E. coli*, respectively. The pMV261-*msmeg_1954* mutants were transformed into *M. smegmatis mc^2^155*, and pGEX-6P-1-*msmeg_1954* mutants were transformed into *E. coli* BL21-Codon Plus (DE3) RIL. The N-terminal truncation of 98 residues (MSMEG_1954N^△^^98^) was constructed into pMV261 and transformed into *M. smegmatis mc^2^155* to obtain pMV261- *msmeg_1954*N^Δ^ ^98^-mc^2^155.

### ATPase Assay

ATPase activity was estimated using QuantiChrom^TM^ ATPase/GTPase Assay Kit (BioAssay Systems). Reactions were performed in 96-well plates in a 40 μL volume with 20 μL of 2 × assay buffer (40 mM Hepes, 80 mM NaCl, 8 mM MgAc2, 1 mM EDTA, pH 7.5), 10 μg of purified MSMEG_1954 protein, or the indicated mutants. Reactions were initiated by the addition of 1 mM ATP. All measurements were performed at 25°C for 20 min for at least three repeats. To test the endogenous ATPase activity, a control with no enzyme was set up under identical conditions. In total, 200 μL of reagent was added to stop the reaction after incubation for 30 min at room temperature. The absorbance at 620 nm was measured using a microplate reader (BioTek, Synergy 4). The rate of ATP hydrolysis was then determined by assaying the liberated Pi and compared with a standard curve. Data were fitted to the standard Michaelis–Menten equation.

## Results

### MSMEG_1954 Is Responsible for Macrolide Resistance in *M. smegmatis*

**FIGURE 1:**
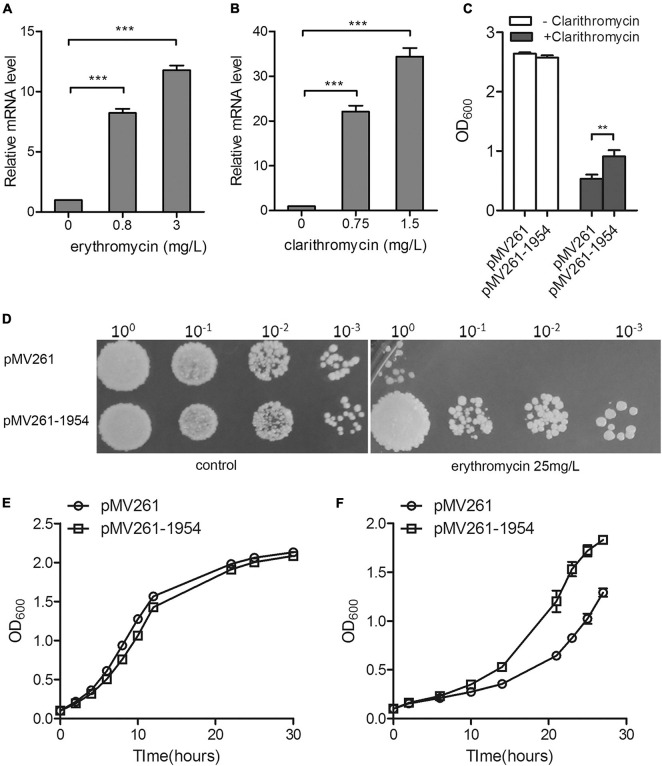
MSMEG_1954 is responsible for macrolide resistance in *M. smegmatis*. The mRNA levels of MSMEG_1954 were increased in response to treatment with erythromycin **(A)** and clarithromycin **(B)**. **(C)** The growth of pMV261-mc^2^155 and pMV261-msmeg1954-mc^2^155 in 7H9 medium with or without 0.75 mg/L clarithromycin. **(D)** The erythromycin resistance phenotype of pMV261-mc^2^155 and pMV261-msmeg1954-mc^2^155. The pictures shown are representative of three independent experiments. **(E)** Growth curves of pMV261-mc^2^155 and pMV261- msmeg1954-mc^2^155 in 7H9 medium without erythromycin. **(F)** Growth curves of pMV261-mc^2^155 and pMV261- msmeg1954-mc^2^155 in 7H9 medium in the presence of 3 mg/L erythromycin (mean ± SD of three individual experiments, **P < 0.01, ***P < 0.001).

To investigate whether MSMEG_1954 is associated with macrolide resistance, we measured changes in the mRNA levels of MSMEG_1954 in response to macrolide treatment in mc^2^155. Quantitative reverse transcription PCR (qRT–PCR) analysis showed that the transcription level of MSMEG_1954 increases upon exposure to different concentrations of erythromycin or clarithromycin (Figures 1A,B).

Next, we constructed strains overexpressing MSMEG_1954 for antibiotic resistance testing. The MSMEG_1954 overexpressing strains (pMV261-1954-mc^2^155) have a growth advantage over *M. smegmatis* that has an empty pMV261 vector (pMV261-mc^2^155) when treated with 0.75 mg/L of clarithromycin (Figure 1C). Likewise, the pMV261-1954-mc^2^155 construct also showed an erythromycin resistant phenotype when compared with pMV261-mc^2^155 strains upon exposure to erythromycin (Figures 1D–F).

Taken together, these results indicate that MSMEG_1954 is induced in response to macrolide exposure and under the overexpression scenario tested here, appears to provide resistance in *M. smegmatis* just like MABP-1 in *Mycobacterium tuberculosis.*

### Two Different Conformations of Apo-MSMEG_1954

**FIGURE 2:**
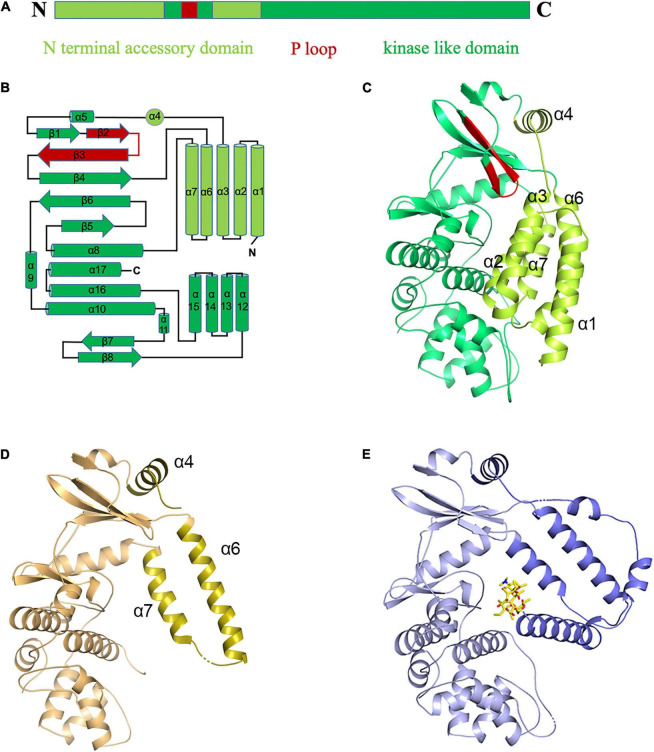
The “closed” and “open” conformations of apo-MSMEG_1954 structure. **(A)** Domain organization of the MSMEG_1954. **(B)** Topology diagram of MSMEG_1954 with the N terminal accessory domain highlighted in limon. The P loop is shown in red. **(C)** The overall structure of MSMEG_1954-form 1. The α helices of N terminal accessory domain in MSMEG_1954-form 1 are tightly packed, which resulted in the closed conformation. **(D)** The overall structure of MSMEG_1954-form 2. The remaining α6 and α7 helices of the accessory domain (olive) are positioned far away from the kinase-like domain, in a way similar to MABP-1. **(E)** The overall structure of MABP-1 with N terminal accessory domain highlighted in slate.

Two crystal structures (form 1 and form 2) of apo-MSMEG_1954 were initially determined (Supplementary Table 1). No electron density for the N-terminal 97 residues (α1-α3 helices) in MSMEG_1954-form 2 was detected. Similar to MABP-1, MSMEG_1954-form 1 and MSMEG_1954-form 2 are both composed of two domains (Figure 2A), the kinase-like domain with a phosphate-binding loop (P-loop), and the N terminal accessory domain that consists of six α-helices: α1–α4 helices (residues 1–114) and α6–α7 helices (residues 158–199) (Figure 2B). The kinase-like domain is conserved in both structures. However, the N terminal accessory domain in MSMEG_1954-form 1 and MSMEG_1954-form 2 have different conformations. More specifically, the N terminal accessory domain (especially helices α1-α3 and α6-α7) of MSMEG_1954-form 1 has a very compact arrangement such that the domain is in direct contact with the kinase-like domain, which block the formation of substrate binding site (Figure 2C). In contrast, the remaining α6 and α7 helices of the N terminal accessory domain in MSMEG_1954-form 2 are positioned far away from the kinase-like domain (Figure 2D), in a way similar to MABP-1 (Figure 2E), which leave the substrate binding pocket open. The SDS-PAGE results of form 2 crystals showed four bands (Supplementary Figure 2), suggesting that the missing N-terminal 97 residues in MSMEG_1954-form 2 were probably degraded during crystallization.

### ADP Induced Conformational Change of MSMEG_1954

**FIGURE 3:**
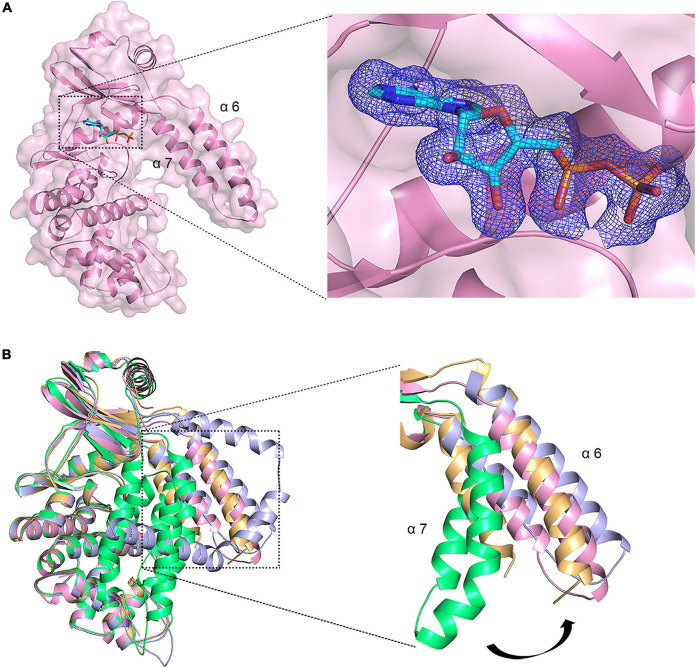
ADP induced conformational change of MSMEG_1954. **(A)** Left, The overall structure of MSMEG_1954 in complex with ADP. The remaining α6 and α7 helices of the accessory domain are positioned far away from the kinase-like domain, in a way similar to MABP-1. Right, the *F*_*o*_-*F*_*c*_ omit map for ADP, contoured at 3.0 σ. **(B)** The structural alignment of MSMEG_1954-form 1(green), MSMEG_1954-form 2 (orange), MSMEG_1954-ADP (pink) and MABP-1(blue). The α6 and α7 helices of the accessory domain undergoes a conformational change in these structures.

To gain further insights into how MSMEG_1954 recognizes ATP, we solved the crystal structure of MSMEG_1954 in complex with ADP. The electron density for ADP was unequivocal (Figure 3A). The density of N-terminal 99 residues was still invisible in this structure. Moreover, structural superimpose show that MSMEG_1954-ADP is similar to MSMEG_1954-form 2 in an open state conformation (Figure 3B). The α6 and α7 helices of the N-terminal accessory domain are positioned far away from the kinase-like domain and fall in a position between the MSMEG_1954-form 2 and MABP-1 (Figure 3B).

**FIGURE 4:**
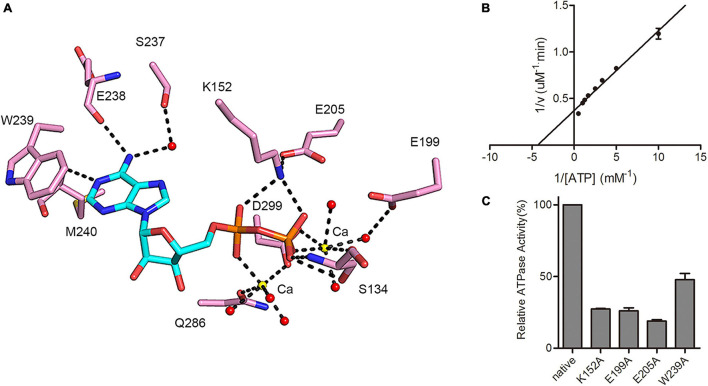
MSMEG_1954 has ATPase activity. **(A)** Residues involved in ADP binding in MSMEG_1954. The ADP molecule is shown as cyan stick, the calcium ions are shown as yellow spheres, the water molecules are shown as red spheres. **(B)** The Lineweaver-Burk plot of ATPase activity of MSMEG_1954. MSMEG_1954 hydrolyzes ATP with a Michaelis constant (*K*m) of 0.231 mM and *k*cat of 0.00864 s^– 1^ for ATP. **(C)** Relative ATPase activities of variants with mutations in the nucleotide binding site (mean ± SD of three individual experiments).

Structural analysis confirmed that the ATP binding pocket of MSMEG_1954 is similar to that observed for MABP-1 (Figure 4A). The adenyl group is held in place by a π-stacking arrangement with the side chain of W239; the N1 atom of the adenyl group forms a hydrogen bond with the carbonyl nitrogen of M240. Hydrogen bonds are also formed between the exocyclic N6 atom of the adenyl group and the carbonyl oxygen of E238. Meanwhile, the side chain of K152 forms hydrogen bonds with the α and β-phosphate group of ADP, the backbone amide of S134 from the P-loop and its side chain form hydrogen bonds with β-phosphate group. The remaining interactions are mediated through the calcium ions which were introduced in the crystallization buffer. One calcium ion bridges the α-phosphate oxygen and the side chain of D299 and Q286, and is also linked to three adjacent water molecules. The second calcium ion is coordinated by the β-phosphate oxygen and D299, along with four adjacent water molecules.

As demonstrated in MABP-1, MSMEG_1954 also has ATPase activity with a *k*cat of 0.00864 s^–1^ and *K*m of 0.231 mM (Figure 4B), values that are comparable to that for MABP-1 (*k*cat = 0.0137 s^–1^, *Km* = 0.285 mM). The typical ABC transporters hydrolyses ATP with a Michaelis constant (*Km)* for ATP of 0.23 mM (Lin et al., 2015), which is similar to MSMEG_1954 and MABP-1. Compared with the wild-type enzyme, the K152A, E199A, E205A and W239A mutants lost 73, 74, 81, and 52% activity, respectively, emphasizing their importance in nucleotide binding and catalysis (Figure 4C). Importantly, all these mutants also have a significantly decreased erythromycin resistant phenotype in *M. smegmatis*, (Supplementary Figure 3), suggesting that antibiotic resistance also depends on the ATPase activity of MSMEG_1954.

### The Macrolide Binding Pocket of MSMEG_1954

**FIGURE 5:**
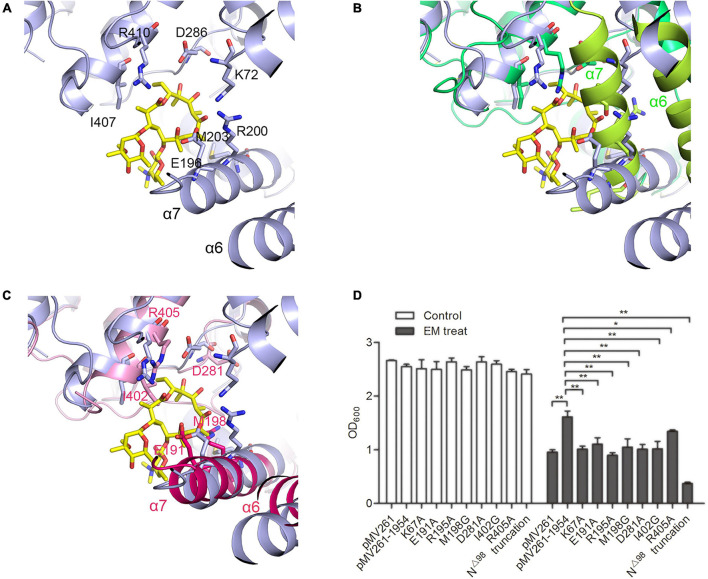
The substrate binding pocketFigure of MSMEG_1954. **(A)** The erythromycin binding pocket in MABP-1 (blue). **(B)** The structural alignment of MABP-1-erythromycin with MSMEG_1954-fom1 (green), the α6 and α7 helices of the N terminal accessory domain are colored as limon. **(C)** The structural alignment of MABP-1-erythromycin with MSMEG_1954-ADP (pink), the α6 and α7 helices of the N terminal accessory domain are colored as hotpink. In order to better display the details of antibiotic binding pocket, all these three structures are flipped compare with Figure 2. **(D)** The growth of mc^2^155 wild type and strains with point mutations in the erythromycin binding site in the presence (+) or absence (–) of 6.25 mg/L erythromycin. [mean ± SD of three individual experiments, *P < 0.05, **P < 0.01)].

Although the sequence alignment studies showed that the substrate binding pocket is conserved between MSMEG_1954 and MABP-1 (Supplementary Figure 1), we have not been able to obtain the structure of MSMEG_1954 bound with erythromycin. Previously, we have described the structure of MABP-1 bound with erythromycin (Figure 5A). The structural alignment of MABP-1- erythromycin and MSMEG_1954 provide details in substrate binding pocket (Figures 5B,C). The substate binding pocket in MSMEG_1954-form 1 is completely blocked by the N-terminal accessory domain. Meanwhile, the antibiotic binding pocket appears to be open in MSMEG_1954-ADP.

A series of mutants containing point mutations in the substrate binding pocket of MSMEG_1954 were made to test their ability to confer resistance to erythromycin. The K67A, E191A, R195A, M198G, D281A, I402A, R405A mutants all significantly reduced the ability of MSMEG_1954 to confer erythromycin resistance when compared with *M. smegmatis* strains overexpressing wild type MSMEG_1954 (Figure 5D), emphasizing their critical roles in binding and in erythromycin resistance.

In order to verify the physiological function of N-terminal domain, the construct with N-terminal 98 residues deleted (MSMEG_1954N^△^^98^) was made. The antibiotic resistance testing showed MSMEG_1954N^△^^98^ completely abolished erythromycin resistance in *M. smegmatis* (Figure 5D), which demonstrates the indispensable function of the accessory domain in substrate binding.

## Discussion

In the previous study, we firstly identified a new macrolide antibiotic binding protein-1 (MABP-1) in the *Mycobacterium tuberculosis*, and verified that it confers inducible resistance to the macrolide family of antibiotics. In this work, we show that MSMEG_1954, the homolog of MABP-1, is responsible for macrolide resistance in *M. smegmatis* strains. The structural studies verified that MSMEG_1954 can assume two different conformations, the closed state (MSMEG_1954-form 1) and the open state (MSMEG_1954_form 2 and MSMEG_1954-ADP). For MSMEG_1954-form 2, the degradation of N terminal 97 residues may decrease the steric hindrance, which release the compact arrangement of N terminal accessory domain. It is likely to represent a transitional state between MSMEG_1954-form 1 and MSMEG_1954-ADP complex structure.

Remarkably, both native MABP-1 and complex structure with ADP or erythromycin have identical conformation, which is similar to MSMEG_1954-ADP, the open state. However, we captured the closed state of MSMEG_1954 (form 1) in this study. As shown in many other works (Rabiller et al., 2010), the crystal structure should always be considered to be a snapshot which depicts the protein at a particular point of a conformational equilibrium rather than be taken as a sole state. It is possible that we failed to capture the closed state of MABP-1, through this protein may have different conformations in physiology.

It should be noted that the inactivation of macrolide has been reported in clinical isolates of *S. aureus*, which can produce macrolide phosphotransferases encoded by mph(C) (Leclercq, 2002). However, the kinase activity was not identified in MABP-1 and MSMEG_1954 despite both of them have conserved kinase domain.

The classical organization of ABC transporters requires interaction between two transmembrane domains (TMDs) which usually impart substrate specificity, and two nucleotide-binding domains (NBDs) which couple ATP hydrolysis to drug efflux, such as MacB (Souabni et al., 2021). However, not all NBD containing protein (NP) harbored a predicted TMD partner(s) in the same transcriptional unit or in the vicinity, such as NPs belong to sub-family 3 in *Clostridioides difficile* (Pipatthana et al., 2021). Interestingly, these NPs exhibited diverse functions from protection against drug targets to the regulation of protein translation (Chen et al., 2014; Murina et al., 2019). Many studies find these proteins are involved in antibiotic resistance, such as methionine sulfoxide reductase [Msr (A)] in *Staphylococci* (Reynolds, 2003) and Msr (D) in *Escherichia coli* (Nunez-Samudio and Chesneau, 2013). The mechanism of antibiotic resistance remains controversial for this kind of ABC proteins, with two competing hypotheses each having gained considerable support: ribosomal protection (Sharkey et al., 2016; Su et al., 2018; de Block et al., 2021) vs. active transport (Nunez-Samudio and Chesneau, 2013; Iannelli et al., 2018).

In our studies, we identify MABP-1and MSMEG_1954 are non-canonical ABC proteins that harbor the nucleotide-binding domains but with no membrane-spanning domains in Mycobacteria, which is similar to sub-family 3 of ABC proteins. There are at least two reasonable possibilities for the mechanism of antibiotic resistance: (i) MSMEG_1954 could bind and dissociate erythromycin from the ribosome in an ATP-dependent manner, reducing the concentration of erythromycin in the cytoplasm that is able to inhibit ribosomal activity. It is like a “sponge” that bind antibiotics to achieve ribosomal protection. We hence name it as sponge hypothesis. However, we have to point out that this suicide action is not an economical method for organisms; (ii) ABC transport hypothesis: It appears possible that they may couple with other efflux pumps to expel the antibiotics from bacterial cells. However, the direct involvement of MSMEG_1954 in the efflux of macrolides remains to be demonstrated. The identification of hypothetical transmembrane proteins that cooperate with MSMEG_1954 is still on going.

**FIGURE 6:**
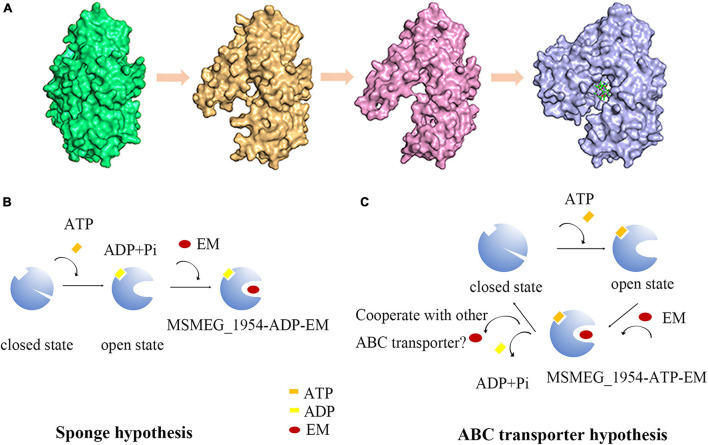
The hypothesis for conformational changes and antibiotic resistance mechanism in MSMEG_1954. **(A)** The structure of MSMEG_1954-form 1 (green), MSMEG_1954-form 2 (orange), MSMEG_1954-ADP (pink) and MABP-1-EM (blue). All these four structures are flipped 180° compare with Figure 2 to better display the antibiotic binding pocket. The diagrams in **(B,C)** show the sponge hypothesis and the ABC transporter hypothesis.

We propose two hypotheses about the relationship between conformational changes and ATP hydrolysis (Figure 6). (i) MSMEG_1954 appears to be the closed state in its apo-form (Figure 6A). Upon ATP binding and hydrolysis, the conformational change occurs from closed state to open state, which enables the binding of antibiotics. The formation of MSMEG_1954-ADP-EM complex reduce the concentration of antibiotics in the cytoplasm and then protect the ribosome from antibiotics binding (Figure 6B). This corresponds to the sponge hypothesis; ii) The conformational change occurs when ATP binding but not hydrolyzing. Hence, ATP hydrolysis is not required for the change from the closed to the open conformations. After antibiotics binding, the MSMEG_1954-ATP-EM complex form. The structural comparison between MSMEG_1954-ADP and MABP-1-erythromycin show that the two ligands are separated from each by only 10 Å (Supplementary Figure 4), with the phosphate moiety pointing directly to the erythromycin. The scenario that ATP-hydrolysis could cause changes in antibiotic binding pocket associated with the release of antibiotics is reasonable. The export of antibiotics likely relies on other ABC transporters that may cooperate with MSMEG_1954 (Figure 6C), which support the ABC transport hypothesis.

## Data Availability Statement

The datasets presented in this study can be found in online repositories. The names of the repository/repositories and accession number(s) can be found in the article/Supplementary Material.

## Author Contributions

ZR designed the research. QZ, HL, and BZ performed the research. QZ, XL, KM, HY, and ZR analyzed the data. QZ, LG, XL, and ZR wrote the manuscript. All authors contributed to the article and approved the submitted version.

## Conflict of Interest

The authors declare that the research was conducted in the absence of any commercial or financial relationships that could be construed as a potential conflict of interest.

## Publisher’s Note

All claims expressed in this article are solely those of the authors and do not necessarily represent those of their affiliated organizations, or those of the publisher, the editors and the reviewers. Any product that may be evaluated in this article, or claim that may be made by its manufacturer, is not guaranteed or endorsed by the publisher.

## References

[B1] Adams P. D. Grosse-Kunstleve R. W. Hung L. W. Ioerger T. R. McCoy A. J. Moriarty N. W. (2002). PHENIX: building new software for automated crystallographic structure determination. *Acta Crystallogr. D Biol. Crystallogr. D* 58 1948–1954. 10.1107/s0907444902016657 12393927

[B2] Alekshun M. N. Levy S. B. (2007). Molecular mechanisms of antibacterial multidrug resistance. *Cell* 128 1037–1050. 10.1016/j.cell.2007.03.004 17382878

[B3] Black P. A. Warren R. M. Louw G. E. van Helden P. D. Victor T. C. Kana B. D. (2014). Energy metabolism and drug efflux in *Mycobacterium tuberculosis*. *Antimicrob. Agents Chemother.* 58 2491–2503. 24614376 10.1128/AAC.02293-13 PMC3993223

[B4] Blair J. M. Webber M. A. Baylay A. J. Ogbolu D. O. Piddock L. J. (2015). Molecular mechanisms of antibiotic resistance. *Nat. Rev. Microbiol.* 13 42–51. 25435309 10.1038/nrmicro3380

[B5] Braibant M. Gilot P. Content J. (2000). The ATP binding cassette (ABC) transport systems of *Mycobacterium tuberculosis*. *FEMS Microbiol. Rev.* 24 449–467. 10.1111/j.1574-6976.2000.tb00550.x 10978546

[B6] Chen B. Boel G. Hashem Y. Ning W. Fei J. Wang C. (2014). EttA regulates translation by binding the ribosomal E site and restricting ribosome-tRNA dynamics. *Nat. Struct. Mol. Biol.* 21 152–159. 10.1038/nsmb.2741 24389465 PMC4143144

[B7] Chen V. B. Arendall W. B. III Headd J. J. Keedy D. A. Immormino R. M. Kapral G. J. (2010). MolProbity: all-atom structure validation for macromolecular crystallography. *Acta Crystallogr. D Biol. Crystallogr.* 66 12–21. 10.1107/S0907444909042073 20057044 PMC2803126

[B8] da Silva P. E. Von Groll A. Martin A. Palomino J. C. (2011). Efflux as a mechanism for drug resistance in *Mycobacterium tuberculosis*. *FEMS Immunol. Med. Microbiol.* 63 1–9. 21668514 10.1111/j.1574-695X.2011.00831.x

[B9] de Block T. Laumen J. G. E. Van Dijck C. Abdellati S. De Baetselier I. Manoharan-Basil S. S. (2021). WGS of commensal neisseria reveals acquisition of a new ribosomal protection protein (MsrD) as a possible explanation for high level azithromycin resistance in Belgium. *Pathogens* 10:384. 10.3390/pathogens10030384 33806962 PMC8005064

[B10] De Rossi E. Ainsa J. A. Riccardi G. (2006). Role of mycobacterial efflux transporters in drug resistance: an unresolved question. *FEMS Microbiol. Rev.* 30 36–52. 10.1111/j.1574-6976.2005.00002.x 16438679

[B11] Emsley P. Lohkamp B. Scott W. G. Cowtan K. (2010). Features and development of Coot. *Acta Crystallogr. D Biol. Crystallogr.* 66 486–501. 10.1107/S0907444910007493 20383002 PMC2852313

[B12] Goldberg D. E. Siliciano R. F. Jacobs W. R. Jr. (2012). Outwitting evolution: fighting drug-resistant TB, malaria, and HIV. *Cell* 148 1271–1283. 10.1016/j.cell.2012.02.021 22424234 PMC3322542

[B13] Goude R. Parish T. (2008). The genetics of cell wall biosynthesis in *Mycobacterium tuberculosis*. *Future Microbiol.* 3 299–313. 18505396 10.2217/17460913.3.3.299

[B14] Iannelli F. Santoro F. Santagati M. Docquier J. D. Lazzeri E. Pastore G. (2018). Type M resistance to macrolides is due to a two-gene efflux transport system of the ATP-Binding Cassette (ABC) superfamily. *Front. Microbiol.* 9:1670. 10.3389/fmicb.2018.01670 30108557 PMC6079230

[B15] Li X. Li J. Hu X. Huang L. Xiao J. Chan J. (2015). Differential roles of the hemerythrin-like proteins of *Mycobacterium smegmatis* in hydrogen peroxide and erythromycin susceptibility. *Sci. Rep.* 5:16130. 10.1038/srep16130 26607739 PMC4660385

[B16] Lin D. Y.-W. Huang S. Chen J. (2015). Crystal structures of a polypeptide processing and secretion transporter. *Nature* 523 425–430. 10.1038/nature14623 26201595

[B17] Livak K. J. Schmittgen T. D. (2001). Analysis of relative gene expression data using real-time quantitative PCR and the 2^–ΔΔCT^ method. *Methods* 25 402–408. 11846609 10.1006/meth.2001.1262

[B18] McCoy A. J. Grosse-Kunstleve R. W. Adams P. D. Winn M. D. Storoni L. C. Read R. J. (2007). Phaser crystallographic software. *J. Appl. Crystallogr.* 40 658–674. 19461840 10.1107/S0021889807021206 PMC2483472

[B19] Leclercq R. (2002). Mechanisms of resistance to macrolides and lincosamides: nature of the resistance elements and their clinical implications. *Clin. Infect. Dis.* 34 482–492. 10.1086/324626 11797175

[B20] Minor W. Cymborowski M. Otwinowski Z. Chruszcz M. (2006). HKL-3000: the integration of data reduction and structure solution–from diffraction images to an initial model in minutes. *Acta Crystallogr. D Biol. Crystallogr.* 62 859–866. 10.1107/S0907444906019949 16855301

[B21] Murina V. Kasari M. Takada H. Hinnu M. Saha C. K. Grimshaw J. W. (2019). ABCF ATPases involved in protein synthesis, ribosome assembly and antibiotic resistance: structural and functional diversification across the tree of life. *J. Mol. Biol.* 431 3568–3590. 10.1016/j.jmb.2018.12.013 30597160 PMC6723617

[B22] Nunez-Samudio V. Chesneau O. (2013). Functional interplay between the ATP binding cassette Msr(D) protein and the membrane facilitator superfamily Mef(E) transporter for macrolide resistance in *Escherichia coli*. *Res. Microbiol.* 164 226–235. 10.1016/j.resmic.2012.12.003 23261969

[B23] Otwinowski Z. Minor W. (1997). Processing of X-ray diffraction data collected in oscillation mode. *Methods Enzymol.* 276 307–326. 10.1016/S0076-6879(97)76066-X 27754618

[B24] Pipatthana M. Harnvoravongchai P. Pongchaikul P. Likhitrattanapisal S. Phanchana M. Chankhamhaengdecha S. (2021). The repertoire of ABC proteins in *Clostridioides* difficile. *Comput. Struct. Biotechnol. J.* 19 2905–2920. 10.1016/j.csbj.2021.05.012 34094001 PMC8144104

[B25] Potterton E. Briggs P. Turkenburg M. Dodsona E. (2003). A graphical user interface to the CCP4 program suite. *Acta Crystallogr. D Biol. Crystallogr.* 59 1131–1137. 12832755 10.1107/s0907444903008126

[B26] Rabiller M. Getlik M. Kluter S. Richters A. Tuckmantel S. Simard J. R. (2010). Proteus in the world of proteins: conformational changes in protein kinases. *Arch. Pharm.* 343 193–206. 10.1002/ardp.201000028 20336692

[B27] Reynolds E. (2003). Msr(A) and related macrolide/streptogramin resistance determinants: incomplete transporters? *Int. J. Antimicrob. Agents* 22 228–236. 10.1016/s0924-8579(03)00218-8 13678826

[B28] Sharkey L. K. Edwards T. A. O’Neill A. J. (2016). ABC-F proteins mediate antibiotic resistance through ribosomal protection. *mBio* 7:e01975. 10.1128/mBio.01975-15 27006457 PMC4807367

[B29] Souabni H. Batista Dos Santos W. Cece Q. Catoire L. J. Puvanendran D. Bavro V. N. (2021). Quantitative real-time analysis of the efflux by the MacAB-TolC tripartite efflux pump clarifies the role of ATP hydrolysis within mechanotransmission mechanism. *Commun. Biol.* 4:493. 10.1038/s42003-021-01997-3 33888866 PMC8062640

[B30] Stover C. K. de la Cruz V. F. Fuerst T. R. Burlein J. E. Benson L. A. Bennett L. T. (1991). New use of BCG for recombinant vaccines. *Nature* 351 456–460. 10.1038/351456a0 1904554

[B31] Su W. Kumar V. Ding Y. Ero R. Serra A. Lee B. S. T. (2018). Ribosome protection by antibiotic resistance ATP-binding cassette protein. *Proc. Natl. Acad. Sci. U.S.A.* 115 5157–5162. 10.1073/pnas.1803313115 29712846 PMC5960329

[B32] Vilcheze C. Jacobs W. R. Jr. (2014). Resistance to isoniazid and ethionamide in *Mycobacterium tuberculosis*: genes, mutations, and causalities. *Microbiol. Spectr.* 2:MGM2-0014-2013. 10.1128/microbiolspec.MGM2-0014-2013 26104204 PMC6636829

[B33] World Health Organization (WHO) (2014). *Companion Handbook to the WHO Guidelines for The Programmatic Management of Drug-Resistant Tuberculosis.* Geneva: World Health Organization (WHO). 25320836

[B34] World Health Organization (WHO) (2016). *Global Tuberculosis Report 2016.* Geneva: World Health Organization (WHO).

[B35] World Health Organization (WHO) (2018). *Global Tuberculosis Report 2018.* Geneva: World Health Organization (WHO).

[B36] World Health Organization (WHO) (2019). *Global Tuberculosis Report 2019.* Geneva: World Health Organization (WHO).

[B37] Zhang Q. Liu H. Liu X. Jiang D. Zhang B. Tian H. (2018). Discovery of the first macrolide antibiotic binding protein in *Mycobacterium tuberculosis*: a new antibiotic resistance drug target. *Protein Cell* 9 971–975. 10.1007/s13238-017-0502-7 29350349 PMC6208485

[B38] Zhang Y. Yew W. W. (2009). Mechanisms of drug resistance in *Mycobacterium tuberculosis*. *Int. J. Tuberculosis Lung Dis.* 13 1320–1330. 19861002

[B39] Zhang Y. Yew W. W. (2015). Mechanisms of drug resistance in *Mycobacterium tuberculosis*: update 2015. *Int. J. Tuberculosis Lung Dis.* 19 1276–1289. 10.5588/ijtld.15.0389 26467578

