# Supplementary material for: Conformational Changes in a Macrolide Antibiotic Binding Protein From Mycobacterium smegmatis Upon ADP Binding
Source: Front Microbiol. 2021 Dec 9;12:780954. doi: 10.3389/fmicb.2021.780954 (PMC8696161; doi:10.3389/fmicb.2021.780954)
Supplement: Supplementary file 1 [file Table_1.docx]

**SUPPORTING INFORMATION**


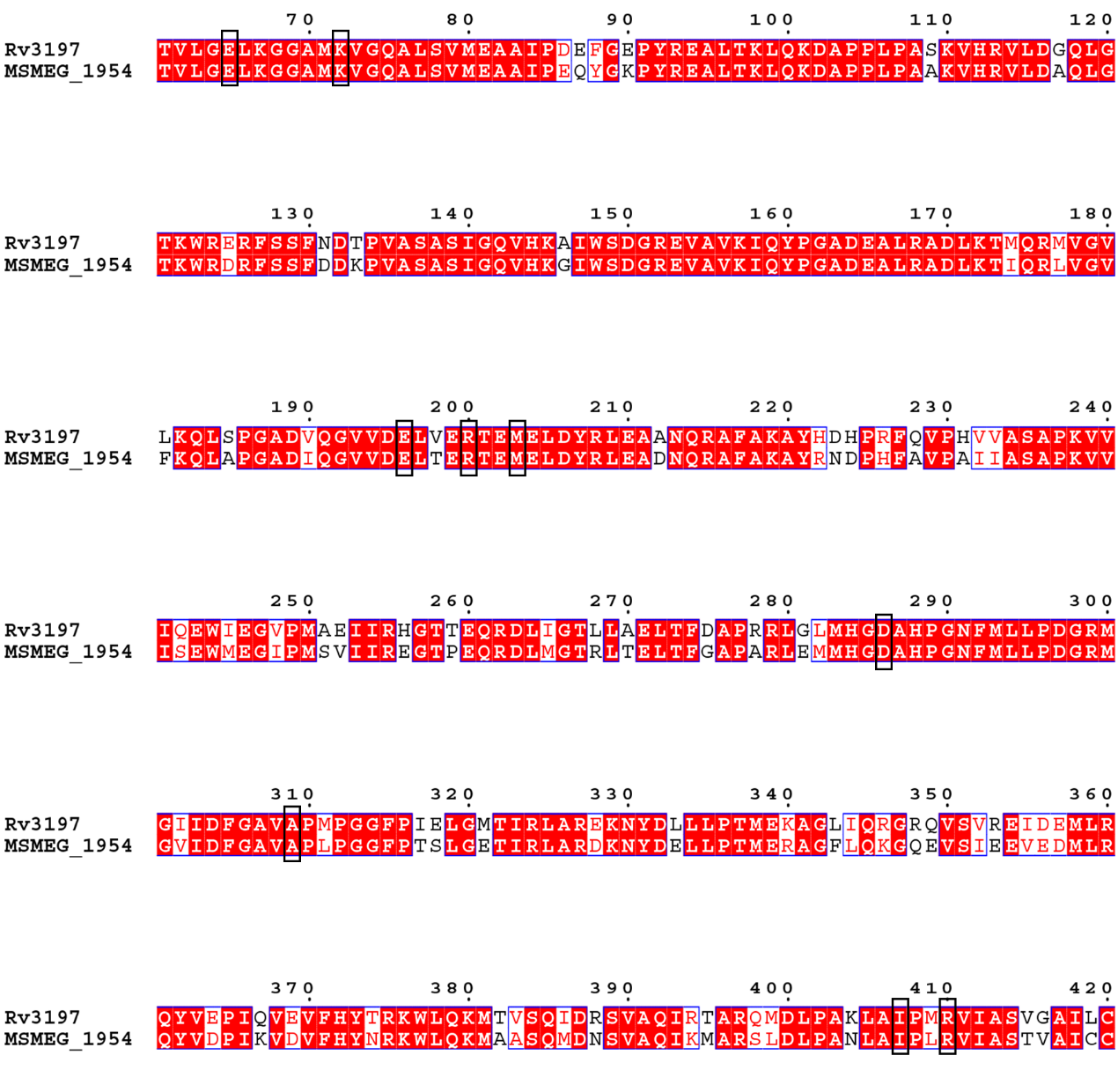


**Supplementary Figure 1. Sequence alignment between MSMEG_1954 and MABP-1. The macrolide binding pocket are conserved between MSMEG_1954 and MABP-1.**


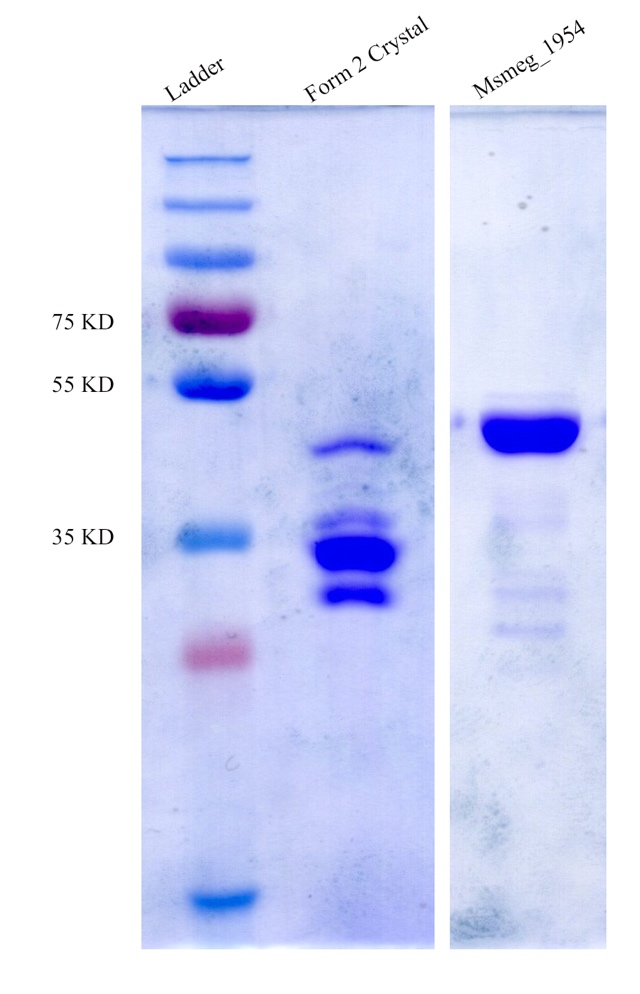


**Supplementary Figure 2. The SDS-PAGE analysis of MSMEG_1954 and form 2 crystals. The molecular weight of MSMEG_1954 is 45 KD**

**
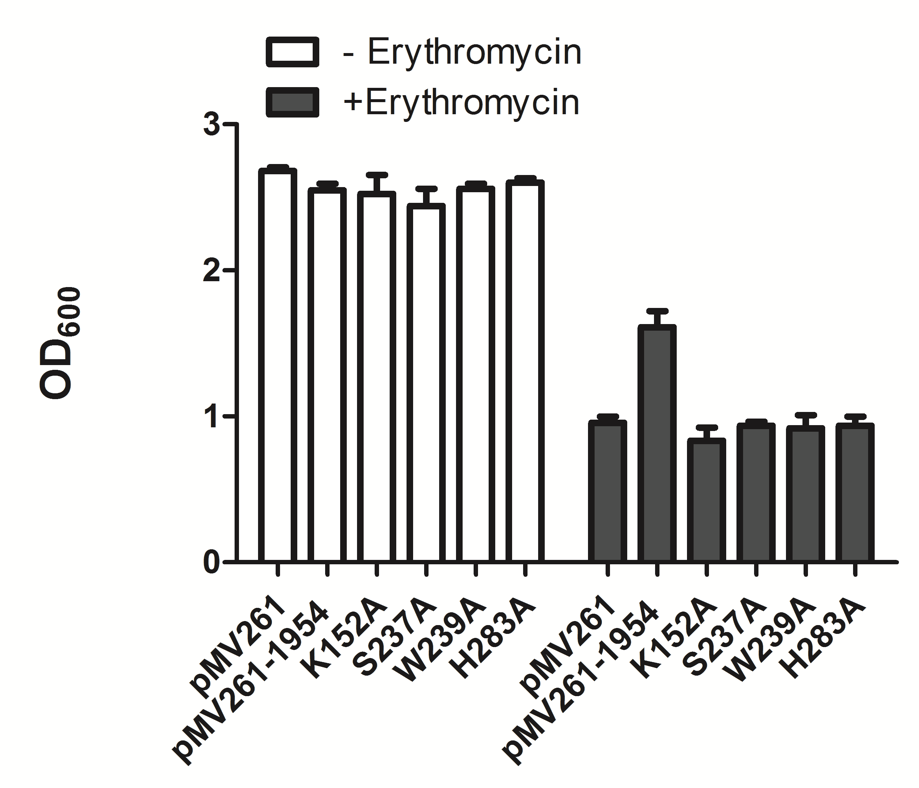
**

**Supplementary Figure 3. The erythromycin resistant phenotype of mutants in ATP binding site of MSMEG_1954 in the presence (+) or absence (−) of 6.25 mg/L erythromycin (mean ± SD of three individual experiments).**

**
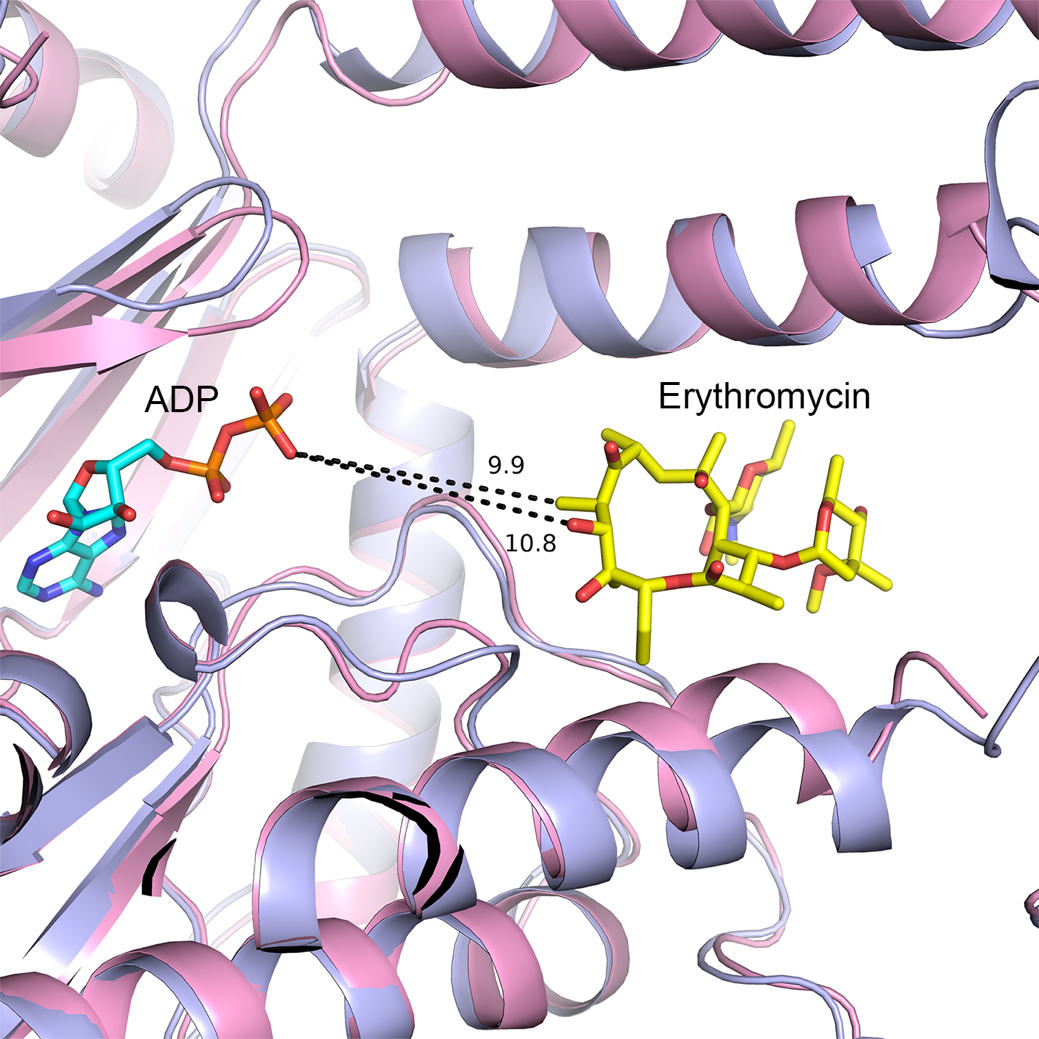
**

**Supplementary Figure 4. The structural superimpose of MSMEG_1954-ADP (pink) and MABP-1-erythromycin (blue). The two ligands are seperated by ~ 10 Å.**

**Supplementary Table 1. Data collection and refinement statistics.**

|  | **MSMEG_1954-**  **Form 1** | **MSMEG_1954-Form 2** | **MSMEG_1954-ADP** |
| --- | --- | --- | --- |
| PDB Code | 7CYR | 7CY2 | 7CZ2 |
| Data collection |  |  |  |
| Space group | *P*2_1_2_1_2_1_ | *P*4_3_2_1_2 | *P*3_2_21 |
| Cell dimensions |  |  |  |
| a, b, c (Å) | 53.6 61.2 117.3 | 74.0 74.0 167. 2 | 83.7 83.7 100.8 |
| ɑ, ß, γ (°) | 90 90 90 | 90 90 90 | 90 90 120 |
| Wavelength (Å) | 0.97916 | 0.97876 | 0.97853 |
| Resolution (Å) | 50-2.05  (2.09-2.05)^a^ | 50-2.75  (2.81-2.75) | 50-1.80  (1.83-1.80) |
| *R*_merge_^b^ | 0.081(0.422) | 0.122 (1.758) | 0.068(1.098) |
| I /σ(I) | 31.8(7.3) | 30.9(2.0) | 30.2(2.4) |
| Completeness (%) | 99.3(100) | 100 (99.9) | 100(100) |
| Redundancy | 13.3(14.0) | 24.5 (21.1) | 9.9(10.1) |
| Refinement |  |  |  |
| Resolution(Å) | 40.32-2.05 | 49.94-2.75 | 38.63-1.80 |
| No. reflections | 24808 | 12163 | 35129 |
| *R*_work_/*R*_free_ ^c^ | 0.179/0.230 | 0.243/0.289 | 0.200/0.231 |
| No. of atoms |  |  |  |
| Protein | 3052 | 2494 | 2430 |
| Water | 293 | 37 | 315 |
| Ligand/ion | 4 | 5 | 33/2 |
| Average B (Å^2^) | 33.0 | 55.0 | 29.0 |
| R.M.S. deviations |  |  |  |
| Bond lengths (Å) | 0.005 | 0.006 | 0.011 |
| Bond angles (°) | 0.970 | 1.115 | 1.737 |
| Ramachandran plot (%) |  |  |  |
| Favored | 98.00 | 98.00 | 96.00 |
| Allowed | 2.00 | 2.00 | 4.00 |
| Outliers | 0 | 0 | 0 |

^a^ Highest-resolution shell is shown in parentheses.

^b^ *R*_merge_ =*Σ_i_ |I_i_-‹I›| / Σ‹I›*, where *I_i_* is an individual intensity measurement and *‹I›* is the average intensity for all the reflections.

^c^ *R*_work_=*Σ ||F_o_|-|F_c_|| / Σ|F_o_|*, where *F_o_* and *F_c_* are the observed and calculated structure factors, respectively.
